# Compliance to infection prevention and control interventions for slowing down COVID-19 in early phase of disease transmission in Dar es Salaam, Tanzania

**DOI:** 10.11604/pamj.2022.41.174.31481

**Published:** 2022-03-04

**Authors:** Janneth Maridadi Mghamba, Ndekya Maria Oriyo, Andre Arsene Fouda Bita, Elizabeth Shayo, Gibson Kagaruki, Reggis Katsande, Ally Hussein, Rogath Saika Kishimba, Loveness John Urio, Nsiande Lema, Neema Camara, Vida Makundi, Tigest Ketsela Mengestu, Grace Elizabeth Saguti, Michael Mesfin Habtu, Elias Kwesi, Muhammad Bakari, Rashid Mfaume, Abel Makubi, Leonard Subi

**Affiliations:** 1 Ministry of Health, Dodoma, Tanzania,; 2 Tanzania Field Epidemiology and Laboratory Training Program, Dar es Salaam, Tanzania,; 3 National Institute for Medical Research, Dar es Salaam, Tanzania,; 4 World Health Organization, Regional Office for Africa, Brazzaville, Republic of Congo,; 5 World Health Organization, Tanzania Country Office, Dar es Salaam, Tanzania,; 6 Muhimbili University of Health and Allied Sciences, Dar es Salaam, Tanzania,; 7 Office of the Regional Administrative Secretary, Dar es Salaam, Tanzania

**Keywords:** COVID-19, Dar es Salaam, Tanzania, mask, physical distancing, handwashing, compliance

## Abstract

**Introduction:**

on 16^th^ March 2020, Tanzania announced its first COVID-19 case. The country had already developed a 72-hour response plan and had enacted three compulsory infection prevention and control interventions. Here, we describe public compliance to Infection Prevention and Control (IPC) public health measures in Dar es Salaam during the early COVID-19 response and testing of the feasibility of an observational method.

**Methods:**

a cross sectional study was conducted between April and May 2020 in Dar es Salaam City. At that time, Dar es Salaam was the epi centre of the epidemic. Respondents were randomly selected from defined population strata (high, medium and low). Data were collected using a structured questionnaire and through observations.

**Results:**

a total of 390 subjects were interviewed, response rate was 388 (99.5%). Mean age of the respondents was 34.8 years and 168 (43.1%) had primary level education. Out of the 388 respondents, 384 (98.9%) reported to have heard about COVID-19 public health and social measures, 90.0% had heard from the television and 84.6% from the radio. Covering coughs and sneezes using a handkerchief was the most common behaviour observed among 320 (82.5%) respondents; followed by hand washing hygiene practice, 312 (80.4%) and wearing face masks, 240 (61.9%). Approximately 215 (55.4%) adhered to physical distancing guidance. Age and gender were associated with compliance to IPC measures (both, p<0.05).

**Conclusion:**

compliance to public health measures during the early phase of COVID-19 pandemic in this urban setting was encouraging. As the pandemic continues, it is critical to ensure compliance is sustained and capitalize on risk communication via television and radio.

## Introduction

The world is experiencing the most devastating health emergency since the 1918 Spanish flu pandemic. The outbreak of coronavirus disease 19 (COVID-19) started in Wuhan, China, in December 2019 [1]. COVID-19 is thought to be primarily transmitted through respiratory droplets with a similar incubation time and generation time as SARS coronavirus (SARS-CoV) [2]. The causative agent was identified as a novel coronavirus, SARS-CoV-2; and the disease is given the name COVID-19. The outbreak was declared a Public Health Emergency of International Concern (PHEIC) by the WHO on 30^th^ January 2020 and declared a pandemic on 16^th^ March 2020. The United Republic of Tanzania had its first imported case detected on 16^th^ March 2020 in Arusha region and subsequently a second case was detected on 18^th^ March 2020 in Dar es Salaam. By mid-April 2020, the disease had spread beyond Arusha and Dar es Salaam, reaching other regions including Zanzibar.

The overarching goal for countries worldwide has been implementing interventions to slow down transmission of the virus and preventing associated illness and death. While initiatives like vaccines and therapeutics are ideal to halt the pandemic, their efficacy of protection differ with existing variants; and outreach, especially in Low-to-Middle-Income Countries (LMICs), availability remains limited. As such, Infection Prevention and Control (IPC) measures, especially non-pharmaceutical interventions, remain to be the most practical and cost-effective interventions which countries have to embark to halt the transmission.

Studies show that adherence to standard IPC precautions (physical distancing, covering mouth and nose with masks, and observing respiratory and hand hygiene), along with enhanced emergency and critical care, and widespread testing and contact tracing, has proven to be effective at slowing COVID-19 transmission [3-5]. Handwashing with soap and water for at least 40 seconds, or use of alcohol-based hand rubs (60-80% alcohol) for at least 20 seconds when hands are not visibly soiled and soap and water are not available, is one of the best defences against the COVID-19 virus [6].

During the early phase of the COVID-19 outbreak, Tanzania adopted a preventive strategy whereby the community was sensitised, and measures were taken to ensure that overcrowding was avoided, and frequent handwashing practiced. A risk communication and community engagement strategy was developed to ensure compliance by the community. Given the above background, we aimed to describe the compliance of the three interventions (hand washing, wearing face masks and maintaining physical distancing) in Dar es Salaam City, which by then was the epi centre of the epidemic. This assessment was also accompanied by a behaviour observational study.

## Methods

This cross-sectional study was carried out during the months of April and May 2020 in Dar-es-Salaam, the largest commercial city in Tanzania, during the time when sporadic cases started to be reported. The total population of Dar es Salaam stands at approximately 6.3 million people. Dar es Salaam is divided into five municipalities (Ilala, Kigamboni, Kinondoni, Temeke, and Ubungo), and the study was conducted in all five municipalities. Study subjects included community members in business areas, bars and bus stands. An initial stratification was done by listing sites with high, medium and low population in each municipality. Random sampling technique was then applied to select study sites and participants in each stratum with variation depending on the number of business areas and bus stands identified in each municipality. For example, 10-15 participants were selected in highly populated site, five in medium and three in low populated sites (Table 1). Lorentz approach was used to calculate the sample size required assuming a 5% level of significance, 5% margin of error, and power of 80%. A total of 390 participants were eventually selected from a total of 40 sites.

**Table 1:** socio-demographic characteristics of respondents, Dar es Salaam, May 2020

Variables	Ilala n (%)	Kinondoni n (%)	Ubungo n (%)	Temeke n (%)	Kigamboni n (%)	Total n (%)
Distribution of respondents	125 (32.1)	77 (19.7)	83 (21.3)	60 (15.4)	45 (11.5)	390 (100)
**Sex**						
Male	59 (47.2)	43 (55.8)	42 (50.6)	27 (45.0)	23 (51.1)	194 (49.7)
Female	66 (52.8)	34 (44.2)	41 (49.4)	33 (55.0)	22 (48.9)	196 (50.3)
**Age**						
Mean (SD)	33.8 (10.3)	33.8 (10.6)	35.5 (12.4)	36.6 (10.9)	35.8 (12.8)	34.8 (11.2)
**Age group (years)**						
<20	4 (3.2)	3 (3.9)	3 (3.6)	2 (3.3)	2 (4.4)	14 (3.6)
20+	121 (96.8)	74 (96.1)	80 (96.4)	34 (56.7)	43 (95.6)	376 (96.4)
**Education**						
None	2 (1.6)	2 (2.6)	3 (3.6)	2 (3.3)	2 (4.4)	11 (2.8)
Primary	46 (36.8)	28 (36.4)	43(51.8)	34 (56.7)	17 (37.8)	168 (43.1)
Not completed primary	2 (1.6)	1 (1.3)	2 (2.4)	2 (3.3)	1 (2.2)	9 (2.3)
Secondary	41 (32.8)	22 (28.6)	17 (20.5)	13 (21.7)	17 (37.8)	110 (28.2)
Not completed secondary	17 (13.6)	7 (9.1)	7 (8.4)	3 (5.0)	3 (6.7)	37 (9.5)
College	17 (13.6)	17(22.1)	11 (13.3)	6 (10.0)	5 (11.1)	55 (14.1)
**Marital status**						
Married	69 (55.2)	34 (44.2)	39 (47.0)	35 (58.3)	23 (51.1)	200 (51.3)
Cohabiting	9 (7.2)	7 (9.1)	6 (7.2)	4 (6.7)	3 (6.7)	29 (7.4)
Single	39 (31.2)	32 (41.6)	33 (39.8)	13 (21.7)	15 (33.3)	132 (33.8)
Separated/ divorced	5 (4.0)	1 (1.3)	2 (2.4)	3 (5.0)	0 (0.0)	11 (2.8)
Widow/widower	3 (2.4)	3 (3.9)	3 (3.6)	5 (8.3)	4 (8.9)	18 (4.6)

Data collection was done using concurrent quantitative and qualitative methods. Quantitative data were collected through interviews using a structured questionnaire while qualitative data were collected through observations. Interviewers and observers were oriented on the tools as well as on the importance of adhering to all COVID-19 preventive measures prior data collection.

Descriptive statistics were summarised by frequencies, percentages, and means with standard deviations where appropriate. We observed minimal missing data of which by rule of thumb does not satisfy the conditions for performing multiple imputation to fix [7]. The observation notes were typed and summarised and converted to numbers so as to get the proportion of people observed complying with the preventive measures specifically hand washing, wearing of face masks and maintaining physical distancing. Triangulation of both data sources was done to enhance validity of the findings. Analysis focused on information sources, and proportion of people practicing public health and social measures. A Chi-square test was used to compare the distribution of participants according to adherence to IPC measures between the categorical variables. All outcome variables included in this analysis were binary, however, some of the explanatory variables were binary thus allowing Pearson Chi-square test for association between outcome and explanatory variable. Other explanatory variables had more than two categories and for such variables, the test was done using Chi-square test for trend. A p-value of <0.05 was considered statistically significant.

Ethical approval for the study was obtained from the Medical Research Coordinating Committee of the National Institute for Medical Research, Tanzania. Permission to conduct the study was also granted by Dar es Salaam Regional Local Government. Written informed consent was obtained from all participants and they were assured of confidentiality, anonymity and voluntary participation. Interviews were conducted in private.

## Results

**Socio-demographic characteristics:** a total of 390 participants were interviewed in all five municipalities with Ilala having 125 (32.1%) participants, Ubungo 83 (21.3%) participants, Kinondoni 77 (19.7%) participants, Temeke 60 (15.4%) participants and Kigamboni 45 (11.5%) participants. The ratio of male to female was 1: 1. Mean age of participants was 34.8 ± 11.2 years and more than 50% were married. Majority of the respondents (43.2%) had completed primary education. Table 1 describes the detailed socio-demographic characteristics.

**Figure 1:**
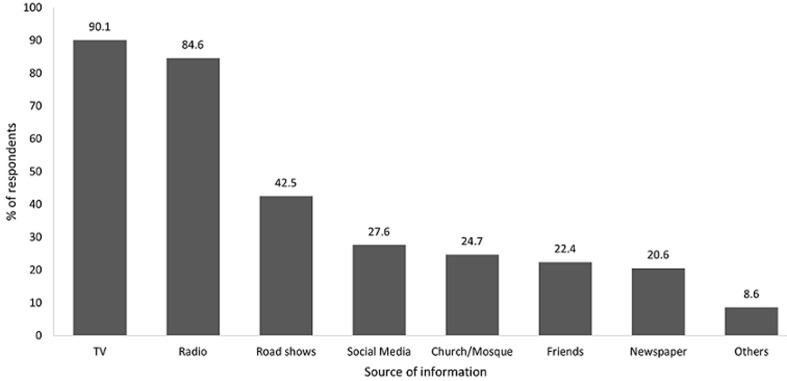
sources of information on COVID-19 prevention measures, Dar es Salaam, May 2020

**Sources of information on COVID-19:** out of the 390 participants, only 2 (0.5%) refused to answer all questions regarding compliance with IPC interventions, making a response rate of 99.5%. Out of the 388 participants who responded, 384 (98.9%) reported that they had heard about public health and social measures related to COVID-19. Majority reported that they got the information through television (90%), followed by radio sessions (84.6%). Other sources included road shows (42.5%), social media (27.6%), churches/mosques (24.7%), friends (22.4%) and newspapers (20.6%). Figure 1 describes in detail the sources of information.

**Figure 2:**
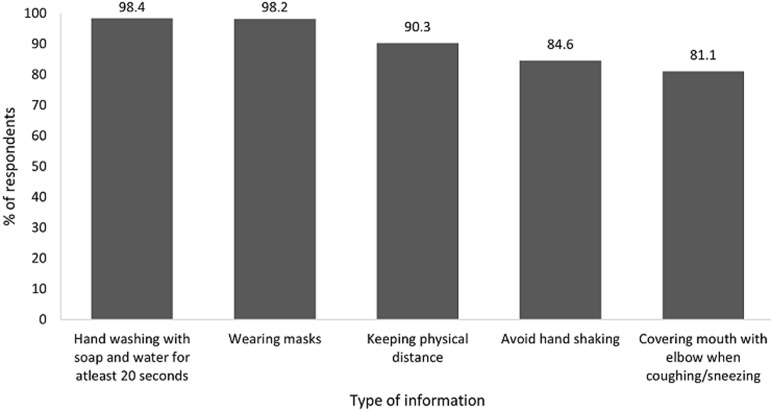
types of information received on key IPC measures to slow down COVID-19, Dar es Salaam, May 2020

Concerning the type of information received, 98.4% reported that they heard about hand washing with soap and water for at least 20 seconds, 98.2% on wearing masks, 90.3% on keeping physical distancing and 84.6% to avoid hand shaking. Figure 2 describes in detail the types of information participants received.

**Hygiene practices during coughing and expelling mucosal fluid (cough etiquette):** during coughing spells, 320 (82.5%) of the respondents reported using a handkerchief, 18 (4.6%) used a piece of tissue paper, 24 (6.2%) used their hands and 26 (6.7%) used part of their clothing to cover their mouths, these results were statistically significant (p=0.037) (Table 2).

**Table 2:** respondent’s hygiene practices when expelling mucosal fluid, Dar es Salaam, May 2020

Cover mouth and nose using	Total, (n=388*)	Ilala (n=125)	Kinondoni (n=75)	Ubungo (n=83)	Temeke (n=58)	Kigamboni (n=45)
Piece of tissue	18 (4.6)	6 (4.8)	4 (5.2)	3 (3.6)	1 (1.7)	4 (8.9)
Handkerchief	320 (82.5)	105 (84.0)	69 (89.6)	68 (81.9)	47 (81.0)	31 (68.9)
Hands	24 (6.2)	5 (4.0)	2 (2.6)	5 (6.0)	6 (10.3)	6 (13.3)
Part of their clothing	26 (6.7)	9 (7.2)	2 (2.6)	7 (8.4)	4 (6.9)	4 (8.9)

* 2 people had missed information

**Figure 3:**
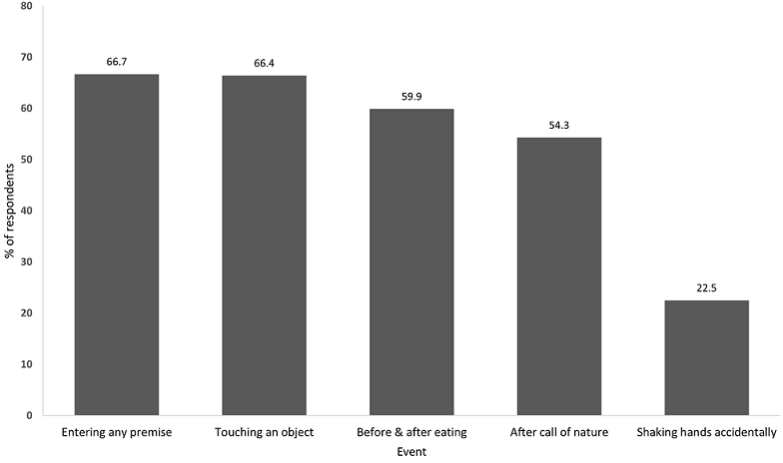
hand washing hygiene practices among respondents, Dar es Salaam, May 2020

**Handwashing hygiene practices:** majority of respondents 382 (98.4%) reported to have been informed on how to effectively wash their hands with water and soap or use alcohol-based gels/sanitizers. When they were asked whether they implemented the recommended practice, 80.4% (n= 312) reported that they did. Out of these, 208 (66.7%) reported handwashing when entering in any premise, 207 (66.4%) and 187 (59.9%) washed hands before and after having meals respectively (Figure 3). Observational results, however, indicated that more than 70% of the population at sites were not doing it correctly (washing for at least 40 seconds), and in some locations, soap was not available. The low populated areas, especially in the supermarkets, people were observed washing their hands correctly using soap and water, whenever they were accessible; while in some of these areas, only sanitizers were provided.

**Practices on physical distancing:** majority of respondents 363 (93.6%) had heard information related to physical distancing. In practical terms, 271 (69.8%) reported that they practiced physical distancing behaviour whenever in a gathering. However, during an observation for about five minutes, only 215 (55.4%) were observed to be practicing physical distancing most of the time when they encountered a large gathering. It was found that reported barriers to observing physical distancing was the lack of sufficient space especially in public means of transport especially buses (*daladalas*) and in business areas when conducting transactions such as listening to customers and convincing customers to buy commodities. These activities are therefore considered risk factors for sustained transmission. It was also found that age played an important role in the practicing of physical distancing with participants in the age group 15-29 seen adhering less than the older participants (Table 3).

**Table 3:** association between non-adherence to the recommended hygienic standards for COVID-19 prevention and demographic factors, Dar es Salaam, May 2020

Variable	Cough etiquette n (%)	Hand hygiene n (%)	Masking n (%)	Physical distancing n (%)
**Gender**				
Males	4 (2.1)	2 (1.0)	65 (33.7)	50 (25.8)
Females	22 (11.3) *	4 (2.1)	80 (41.5)	67 (34.5)
**Age group (years)**				
15-29	6 (4.0)	1 (0.7)	66 (43.7)	57 (37.5) *
30-49	14 (7.3)	4 (2.1)	66 (34.4)	55 (28.7)
50+	6 (13.6)	1 (2.3)	13 (30.2)	5 (11.4)
**Marital status**				
Married/cohabiting	15 (6.6)	3 (1.3)	72 (31.7)	63 (27.5)
Not married	7 (4.9)	3 (2.1)	67 (46.9)	50 (35.0)
**Education**				
None/primary	17 (7.6)	5 (2.3)	84 (37.7)	76 (33.9)
Secondary	8 (7.3)	1 (0.9)	43 (39.5)	29 (26.4)
College	1 (1.9)	0 (0.0)	18 (33.3)	12 (22.2)
**Municipal**				
Ilala	9 (7.2)	0 (0.0)	31 (25.2)	59 (47.2) *
Kinondoni	2 (2.6)	4 (5.3) *	25 (32.5)	22 (28.6)
Ubungo	7 (8.4)	0 (0.0)	47 (56.6)	23 (27.7)
Temeke	4 (6.9)	0 (0.0)	22 (37.9)	7 (12.1)
Kigamboni	4 (8.9)	2 (4.4)	20 (44.4)	6 (13.3)

* P-value <0.05

**Figure 4:**
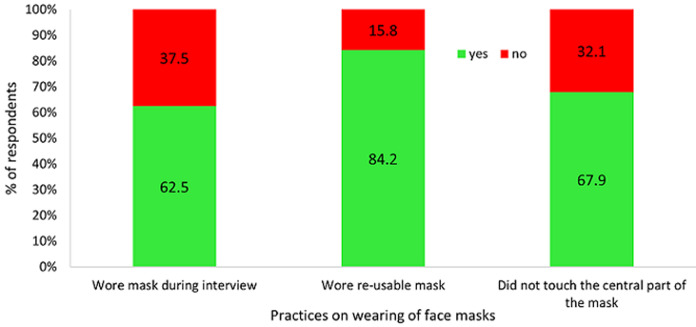
proportion of respondents who were found wearing masks during the interview, Dar es Salaam, May 2020

**Practices on wearing of face masks - observational assessment:** out of the 388 respondents, 382 (98.4%) reported to have been informed on how to wear face masks. During the observational assessment, 240 (61.9%) of those observed were found to be wearing face masks of whom, 150 (62.5%) wore masks correctly, 77 (32.1%) kept on touching the central outer layer of the mask during the interview without sanitizing their hands and 202 (84.2%) wore re-usable masks (Figure 4).

Furthermore, 120 (50.0%) of 240 participants who were wearing masks, reported reasons for wearing the respective type of mask they had on whether disposable or re-usable; 61 (50.8%) reported price, 61 (25.4%) comfort and 31 (12.9%) described their masks as user-friendly to wear and speak with. However, of the 148 respondents who did not wear masks, 78 (52.7%) felt they did not need them, and they could fight the disease with their immunity; 37 (25.0%) claimed that they could not breathe properly with masks on, 21 (14.2%) felt they made them hot and 12 (8.1%) were tired of wearing them.

## Discussion

The results of this study show that residents of Dar es Salaam were largely aware and were practicing IPC interventions for COVID-19 control. More than half of the people observed used proper respiratory etiquette, as well as wearing masks and practicing physical distancing. There was slight variation among municipalities although this was not statistically significant. It was shown that age and sex played an important role in the practicing of respiratory etiquette and physical distancing as selected public health measures with females seen adhering more than males.

The main source of COVID-19 information for the respondents in this study were television and radios. This is also consistent with a study done in Vietnam, where radio and television contributed to 71% of information on COVID-19 [8], whereas religious sites were the source for 32% of the respondents. These findings highlight the importance of using these channels in promotion of IPC measures for COVID-19. This study found that almost all those interviewed had heard of IPC measures to slow down COVID-19 transmission. Similar results have been reported by Qi Y *et al*. (2020) [8]; but in their setting, study subjects reported to have obtained health information mainly through WeChat-a social media platform or websites, whereas in this study it was through radio and television. Despite the variation on channels of communication, in both settings, it is evident from the studies that community are able to adopt health messages for prevention of COVID-19.

This study has shown that more than three quarters of people living in the municipality of Dar es Salaam do cover their mouth when coughing and sneezing. This was also noted in a study done in the Municipalities of China during the COVID-19 pandemic, where more than three quarters of study subjects covered their mouth when coughing or sneezing [8]. The fact that in this study, compliance to the preventive measures was high, this links also to the findings in this study that many people had knowledge of COVID-19 IPC measures. The results of this study are encouraging as COVID-19 is mainly transmitted through large respiratory droplets which has also been supported by recommendations by the US Centers for Disease Control and Prevention (CDC) on prevention of spread of respiratory pathogens [9]. On the other hand, we acknowledge a limitation when reporting the effectiveness of these health messages, due to absence of baseline information.

The proportion of those wearing face masks was still sub-optimal as only 60% wore them correctly. This finding is similar to studies done by Wang HT *et al*. (2020) and Michael HH *et al*. (2020), where they found the proportion of people wearing masks before becoming illness was low [10, 11]. Many studies have documented the role of face masks in slowing the spread of COVID-19 [12-14]. Others have shown that wearing of masks are effective when used in combination with frequent hand-cleaning with alcohol-based hand rub or soap and water [15]. It should be noted that in countries such as Taiwan, Hong Kong and South Korea, a combination of approaches to slow down COVID-19 disease has been used and has been successful [16,17].

This study found a high proportion of participants were practicing hand washing. This shows that there was high acceptance of this measure among the community and has been observed also in many other countries where “no-touch” hand washing gadgets have been innovated e.g. in Kenya, Singapore, India, Australia and New Zealand, and their governments encouraged people to practice effective hand hygiene with soap and water or regularly sanitize with alcohol-based hand rubs in public settings [18-20]. Findings in selected municipalities of China, also found high acceptance of handwashing [21]. The study, however, found that, more than 70% of those who washed their hands, were not doing it correctly. Similar results were also found among medical imaging professionals in India, where there was also lack of knowledge on steps involved in hand washing [22].

Some studies have documented the importance of physical distancing measures for controlling spread of influenza pandemics [2,23]. This study found that a low proportion of people were complying with this measure. Kishinchand *et al*. 2020, observed that it is difficult to adhere to physical distancing practices in the urban poor, particularly those living in slums with limited space. Some of these behaviours related to adherence may be associated with the educational and income level of inhabitants of these areas. This study found however, a higher level of adherence to IPC practices in areas which are known to have lower density and higher incomes. It should be noted that WHO, US CDC and Africa CDC recommend that physical distancing measures should be maintained for prolonged periods [24,25].

A particular strength of this study is the addition of an observational component which recorded what people actually do and this assisted in triangulation of results, this is especially good as it reduces desirability bias. The fact that this study was done in general population and not in well-established institutions like hospitals, gives it strength in terms of generalization of the compliance uptake results. This study however, has few limitations. The study only evaluated compliance and not effectiveness and would be beneficial if effectiveness would be linked with compliance. Moreover, as a cross-sectional study it was not possible to describe the regularity of follow-up of the three barrier measures by participants. Additionally, as with any observation technique, observer bias cannot be overruled.

## Conclusion

As the world continues to put mitigation measures against the virus that causes COVID-19 and its new variants, some of which are presumed to be more transmissible and more virulent, IPC interventions mainly non-pharmaceutical will continue to play a critical role in controlling the spread of COVID-19. Health authorities should not neglect advocating for these measures among the general public but also should strive to evaluate their interventions on COVID-19 response.

### What is known about this topic

- *Preventive measures such as educating the public about the nature of the disease, mode of transmission, physical distancing, hand washing, and wearing of face mask have been proven to be vital for slowing down COVID-19 transmission;*
- *Compliance to non-pharmaceutical interventions by community has been a challenge especially in urban setting*.

### What this study adds

- *This is the first study that reports information from Tanzania on COVID-19 response efforts;*
- *This study has identified public transport and business areas as areas for increased COVID-19 transmission, this information informs national and international response efforts in similar settings;*
- *The unique study methodology used demonstrates the value of including observations to complement interviews when drawing conclusions*.
